# Traditions, Paradigms and Basic Concepts in Islamic Psychology

**DOI:** 10.1007/s10943-018-0595-1

**Published:** 2018-03-23

**Authors:** Rasjid Skinner

**Affiliations:** 1 Institute of Clinical Psychology, University of Karachi, Karachi, Pakistan; 2 Department of Clinical Psychology, University of Sheffield, Sheffield, UK; 3 Ihsaan (Islamic Psychological Therapy Service), Bradford, UK; 4 Cambridge Muslim College, Cambridge, UK

**Keywords:** Islamic psychology, Paradigm, Concept

## Abstract

The conceptual tools of psychology aim to explain the complexity of phenomena that psychotherapists observe in their patients and within themselves, as well as to predict the outcome of therapy. Naturally, Muslim psychologists have sought satisfaction in the conceptual tools of their trade and in what has been written in Islamic psychology—notably by Badri (The dilemma of Muslim psychologists, MWH London, London, 1979), who critiqued Western psychology from an Islamic perspective, arguing the need to filter out from Western Psychology which was cross-culturally invalid or was in conflict with Islamic precept. In this paper, I advocate an extension of Badri’s (1979) approach and present a working model of the self derived from traditional Islamic thought. This model, though rudimentary and incomplete, I believe, makes better sense of my perceptions as a clinician than any other psychological model within my knowledge.

## Introduction

The genesis of this paper goes back to my own discomfort as a practising psychologist with the conceptual tools of my trade. Even though by the grace of God my patients have generally improved, I was never wholly able to explain the outcome of my therapy, nor did I feel that the range of psychological theories of which I had knowledge were adequate to explain the complexity of phenomena I observed in my patients or experienced within myself. It was natural that as a Muslim I should have sought satisfaction in what had been written of Islamic Psychology—notably by Badri (1979) and others of his school, but what I found, though useful, I did not feel adequate for my needs. What did occur over the years, partly through my general study of Islamic culture and from discussions with traditional therapists, notably Hakim Salim Khan, was a working model of the self derived from traditional Islamic thought. This model, though rudimentary and incomplete, I believe, has made better sense of my perceptions as a clinician than any other model within my knowledge.

## An Islamically Based Model of Human Psychology

Over the last couple of years, I have become increasingly aware that other Muslims studying psychology and related disciplines have also experienced a dissonance between what they have been taught from “Western” tradition and their own sense of what is right and real—but without being able to articulate precisely where the dissonance lies.

It therefore seemed appropriate to put my own groupings towards an Islamically based model of human psychology into a coherent form and to seek the collaboration of other Muslims, with an interest in the same field, in improving our collective understanding.

It is proper to consider in which sense one can use the term “Islamic Psychology”. There is a view that psychology should be regarded as a natural science (in the Western definition) concerned with objectively verifiable human behaviour—and, as such, is compatible with Islam and open to use by Muslims. This is essentially part of Badri’s position (Badri 1979).

To Badri, the first task of the Muslim psychologist is to work within the empirical tradition and to distil from the Western corpus of psychology its culturally odourless, experimentally sound, objective components (such as Behaviourism)—rejecting the non-empirical hotchpotch of myth and unproven value-laden theory (such as much of Psychoanalysis) which contains Islamically antagonistic elements.

The second task of the Muslim psychologist is then to ensure that the science is used in accord with Islamic precept. Thus, behavioural principles can be used to treat a sexual dysfunction between a man and his wife, but not to facilitate adultery. In Badri’s definition, Islamic psychology is essentially sound empirical psychology used morally.

Badri has, in my view, valuably identified to Muslims the danger of swallowing wholesale the Western corpus of psychological science. What I found surprising was that Badri—in constructing an Islamically acceptable alternative—largely ignored the classical tradition of Islamic thought in this area, perhaps because it does not always fit into the Western scientific logical positivist mould, which, while doubtless non-toxic to Muslims, lacks those hallmarks of Islamic science (and *ipso facto* all good science)—comprehensibility and explanatory power.^1^

We can take an analogy from architecture and ask “Is this building^2^ Islamic?”. The structure is not falling about our ears so has doubtless been constructed on sound empirical principles. It is also, by housing an Islamic conference, arguably being used to good purpose. Yet, clearly, this building is not Islamic in the sense that The Dome of the Rock or the Arab merchant houses of the Zanzibar waterfront are Islamic. A definition of Islamic architecture couched in terms of the coarse empiricism of its engineering principles is insufficient. When we analyse traditional Muslim architecture, we can discern that its distinctiveness depends on certain underlying schemas determining the way in which sound construction principles are organised. For example, in the creation of balance in proportion and shape, in the unity of design within a diversity of decorative form, in the organic relationship of the building to natural features of sun, wind, water and so on. These themes of balance, unity in diversity, the organic interrelatedness of the created world run throughout the *Qur’an*, yet it is hardly conceivable that early Islamic architects sat round together and intellectually devised these rules of design from a study of primary Islamic sources (i.e., the *Qur’an* and prophetic traditions). It is more plausible that the Islamic environment in which theses early architects were immersed, naturally and unconsciously formed the paradigms which directed their creativity.

What applies to architecture can apply to all science. Kuhn’s (1964) thesis demands respect, that science does not evolve within a vacuum but within a cultural milieu, a paradigm, which influences the way the scientist perceives the world, the questions he asks, his methodology and the way he perceives and gives meaning to the pattern of empirical data. It is certainly possible to speak of Islamic physics, chemistry, medicine, mathematics, psychology and so on, when these sciences have evolved from Islamic paradigms and are distinguished as such. Psychology, moreover, stands apart from the natural sciences as being even more susceptible to a distinctly Islamic imprint. This is partly because the *Qur’an* speaks specifically of the nature of man and of the component parts of the self, partly because the nature of the internal psychological processes makes them not susceptible to objective experimental study. Psychology is thus much more dependant on “models”, or myths in the Jungian meaning, which cannot be proven or disproven in a logical positivist sense, but which have a validity dependant on how good or bad they are at enabling the individual to make sense of internal experiences. Psychoanalysis needs to be given credence as, however imperfectly, its models and vocabulary enable complex mental phenomena to be described. But psychological models which are not made dependant on experimental proving are open to being particularly coloured by the cultural paradigm of their generators and also by culturally determined characteristics of the population studied. A neat illustration of this last point is provided by Personal Construct Theory which claims to describe a universal model of how man gives meaning to his world. I pointed out to a devot of this theory that Construct Theory seemed to me to describe most aptly the thought characteristics only of Western Protestants. My colleague pondered this statement and then admitted that he had been surprised when attending an International Conference of Construct Theorists, that the majority of delegates hailed from the American Bible Belt. And it is of course a commonplace observation that Freud’s “universal” theory, based on observation and practice amongst the “Victorian” middle-class Viennese, loses validity the further one moves from that historical period and culture.

If psychology is permeated with specifically “Western” cultural influences, it is proper to be sceptical about the universality of its statements. If, however, one is to construct a soundly based psychology that harmonises with Islamic thinking, it is better to begin, not by taking Western psychology and attempting to reduce it to its empirical parts, but by going back to the *Qur’anic* statements on the self and the way these statements were constructed into practical models by those classical scholars such as Al-Ghazali, Ibn Sina and Ja’far As-Sadiq who operated fully within an Islamic paradigm (this is the approach adopted by Karim 1984 using the model of Shafii 1985). Karim’s paper, however, I feel does not adequately demonstrate the explanatory power of his adopted model; Shafii (1985) is largely confined to a comparison between his Islamic model and Psychoanalysis.

## A Working Model of the Self Derived from Traditional Islamic Thought

The *Qur’an* refers to three aspects of the self: *Qalb*, *Aql* and *Nafs*)—the last of which can be expressed in three types or levels: *Nafs Ammara*, *Nafs Lawwamma* and *Nafs Mutmaina*. These terms have a wide and subtle range of meaning. One reason for not attempting to construct a “psychology” simply with reference to the *Qur’an* is that an understanding of how these terms can be applied in clinical practise requires such a combination of *Qur’anic* knowledge and clinical experience, operating from an Islamic paradigm of thought, that I doubt today that it can be achieved. There is thus a strong argument for taking account of the use made of such terms in the models constructed and applied by classical Muslim scholars/physicians/psychologists. These terms and their underlying concepts are used variably by traditional scholars. I therefore have taken what I believe to be the more general interpretation, substantially following Khan (1986), but see also Schimmel (1964).

*Qalb* is seen as the isthmus (vide: Stoddart 1976) which connects the self with *Ruh* (the spirit). It is that centre in which the consciousness of God resides: the part of human being in which is felt the sense of *Fitrah* (what is natural) and the inherent sense of right and wrong, and is held to be capable of receiving inspiration.

*Aql* can be defined as the reasoning centre, intelligence, which ordinarily comprehends and analyses external data and can articulate and according to Al-Ghazali (ref.) connects directly with the *Qalb*.

Nafs can be best defined as “life force”. For psychological purposes, in their lower form, they can be regarded as instinctual drives or energies which have a semi-autonomous life of their own.

In themselves, the action of *Nafs* would operate according to *Fitrah* (as with animals), but in human beings they are inherently vulnerable to disruption resulting from, for instance, dysfunctional cognitions, and the effects of emotional traumata, which pervert their operations.

In the ideal, healthy, balanced person, consciousness is centred in *Qalb* which is open to *Ruh* and which directs *Aql*, and together with *Aql* directs the lower *Nafs*. In this state, *Aql* is able to exercise sound reason, its perception of the external world is realistic, it sees the value of moral conduct, its operations are imbued with wisdom, and it is able to exercise a wholesome direction on the *Nafs*. The disrupting influences on the *Nafs Ammara* (the lower *Nafs*) is weakened, and their tendency to wrong action countered by the *Nafs Lawwamma* (the instinct to remorse). And from this state, the self is capable of being transformed, even if only temporarily, into the *Nafs Mutmaina* (the tranquil or rested *Nafs*), which could be described as the state in which the self is in total surrender to the *Qalb* and completely incorporated into the worship of God.

Classically, the analogy often given to describe the relationship between the *Qalb*, *Aql* and *Nafs* (vide: ref.) is that of a rider on their horse (Al Ghazzali 1962). The balanced person is like the rider whose horse takes him where he wants to go, and where he wants to go is in accord with the will of God. Various permutations of imbalance can be illustrated from the basic analogy, for instance, the case of the horse being reined in or hamstrung and going nowhere, the horse out of control and “riding” the rider to destruction, the horse under control but being directed the wrong way.

The Muslim understanding of instinctual drives (*Nafs Ammara*) is subtly but crucially different from that pertaining in Christian culture. In the Islamic view, these drives—though having a vulnerability to act destructively and lead the self in spiritual darkness—are none the less regarded as an indispensable part of the Self which the individual is required to keep in a healthy active state in order to fulfil his life in this world. The emphasis therefore is on the control and transformation of these forces to the best level of functioning, not on their suppression or extinction. In the Pauline Christian view however, instinctual drives are viewed more negatively, with suppression often encouraged. It may be that nowadays, in the West, few people pay much attention to St. Paul, but a negative attitude to instincts such as sexuality and aggression remains endemic in Western society with the consequent problems of internal tension, guilt, conflict and suppressed energy. Universal theories of psychodynamics based exclusively on Western population are therefore inevitably culturally skewed.

It seems to me that at least one major advantage of the Islamic model of the relationships between the *Qalb*, *Aql* and *Nafs* is that it permits a definition of mental health which is independent of socially defined notions of normal and abnormal and which truly can be applied universally. It also points to the diagnosis of pathology and to corrective therapeutic action.

One can suppose that one effect of a truly Islamic cultural environment is to make it easier to maintain a state of God-consciousness and harmony within the self. Education based on *Wahy* (revelation), the *shari’ah* legal system, the acts of *Ibadat* (worship), Islamic art, the dietary rules, the prevalent social mores serves to facilitate the *Qalb*’s purification and openness to *Ruh*, the wholesome channelling of the energy of the lower *Nafs*, and the harmonisation of the thinking process with the intuition of the *Qalb*.

Both *Qur’an* and *Hadith* say of the *Qalb*, however, that it can become sealed (*Qur’an* 2:7) or hardened (*Qur’an* 2:74). It can be supposed that this occurs when, ultimately through man’s choice, the *Qalb* ceases to be exercised within *dhikr* (God remembrance) and/or the *Aql* and *Nafs* become disassociated from its direction through external influence. *Nafs* can become unruly through bad social conditioning or inappropriate diet and inflame *Aql* with inappropriate fears—or cause it to rationalise (in the Freudian sense) actions that are emotionally impelled. *Aql* can be taught to reason from premises not in accord with *Fitrah* (as the Prophet is reported to have said “some reasoning is like sorcery”), produce a distorted conscience and enter its own conflict with the *Nafs*—with psychopathological consequences of which we are probably all aware. A partial dissociation from the *Qalb* by the *Aql* can produce a sense of loss and anomie.

It would be beyond the scope of this paper to describe the effects of every permutation of imbalance possible with this model. But it should I think be clear that it allows a more comprehensive and convincing system of diagnosis than is so with most Western theories. We can example this point by a comparison with Psychoanalysis. Freud’s concept of Libido approximates to that of *Nafs*, but as Psychoanalysis makes no recognition of *Qalb* there is no understanding of the power of the heart to transform the *Nafs*—yet this process can be a matter of personal experience.

For Freud, the dynamics of the self are determined by the generally bellicose relationship between Ego (*Aql*?), Id (*Nafs*?) and Super-Ego. It is tempting to translate Super-Ego (the unconscious conscience) as *Dhameer*, but *Dhameer* is a richer concept than Freud’s view of conscience. In psychoanalytical thought, the Super-Ego is formed through social learning and is inherently in opposition to the will of the Id: its values are culturally relative.

In the model, I have presented conscience (*Dhameer*) as a product of the converging influences of *Aql*, *Qalb* and *Nafs* (*Lawwamma*). In the healthy man, these three forces harmonise with and reinforce each other—the intrinsic feeling of the *Qalb* of what is right and moral, meets with the moral learning of the *Aql* and is reinforced, following wrong action, by the sense of remorse from the *Nafs Lawwamma*. *Dhameer* in turn reinforces moralistic thinking and the capacity for remorse and helps “keep open” the heart. If Psychoanalysis is (at best) less complete as a theory of human psychology than the traditional Islamic model, most other Western theories compare even less favourably when the width of their explanatory power is considered. This is not to say that the observations made within the limited focus of any particular “Western” theory are not of use to the Muslim practitioner. Badri (1979) cannot be faulted in his assertion that the laws of behavioural psychology have validity and in that sense can be considered Islamic. A whole doctoral dissertation, I have been told, has been written on Al-Ghazali’s use of behavioural principles in treatment. For Al-Ghazali, however, Behaviourism was only one weapon in his therapeutic armoury, and he used its techniques within an Islamic model—principally to train disordered forces in *the Nafs Ammara*. The Western psychological theory that shares the most with the traditional Islamic one is probably that of CG Jung—a view which as support from Karim (1984). Jung’s notion of the Depth Unconscious (1961) as containing knowledge of God, and an instinctive wisdom that could guide and balance the rest of the self comes close to the Islamic understanding of *Qalb*. Likewise, Jung’s view (1961) that the principal aim of therapy was to open up the self to the guidance of the Depth Unconscious corresponds surprisingly well to the aim of opening up the *Qalb* in Islamic therapy. There are various other points of Jungian psychology in both theory and application which fit into an Islamic schema, but a proper comparison would well justify a paper in itself.

The weaknesses of Jungian psychology are, however, that: (1) even in its simplest reduction it lacks the simple clarity of the Islamic model, and (2) perhaps inevitably the understanding of the self and its dynamics is skewed by the particular characteristics of the European psyche studied by Jung. The Shadow Archetype of Jungian theory, I have, for instance, found expressed in my Christian and Jewish patients, but never significantly so in my Muslim patients. This difference presumably reflects different cultural/religious attitudes to *Nafs*.

## Therapeutic Implications of the Islamic Model

I would like to turn next to consider briefly the therapeutic implications of the Islamic model. A characteristic of all traditional Islamic science is its perception of the created world as being interconnected in its parts and constituting an essential unity or wholeness. The model I have presented in this paper does I think describe man as a whole, and it enables the self to be related both to the spiritual world and to the physical and social environment. The traditional Islamic approach to therapeutics can likewise be described as wholistic. If we take a common neurotic category of anxiety state, the condition can be dealt with by: (1) medicines and dietary control to weaken the primary *Nafs* of anxiety, (2) behavioural method to train the anxiety *Nafs*, (3) depth analysis and cognitive methods to correct unnatural thought and to open the *Aql’s* connection the *Qalb.* Both aims if achieved help *Aql* to establish some control over the *Nafs*, (4) *dhikr* and other acts of worship can be used to open the heart and extend its control over the imbalances in the *Aql* and *Nafs*.

The emphasis is given to each approach being dependant on a more subtle diagnosis of the dynamics behind the condition.

It can be seen that the Western corpus of psychological knowledge need not be disregarded, what is important for the Muslim is that he has the basic Islamic understanding of the self to enable him to make the right selection from that knowledge and to use it in a harmonious way.

There are, however, areas of therapy which are part of the Islamic tradition, of looking at man in total and in relationship with his environment, but which are largely disregarded in Western psychology: the use of music, for instance, and of environmental psychology in treatment. Ibn Sina (vide: Tibbs 1981) used colour in the treatment of emotional disorders, and I was once told by a German Engineer of a sixteenth-century asylum in Iran he had visited which produced tranquilising effects on severely disturbed psychotic patients by the use of proportion, shape, colour (predominantly blue) and running water in the design of the building.

It is also important to note that any particular treatment modality (for example physical medicine) can have its own tradition developed from within an Islamic paradigm.

## Conclusion

In this paper, I have suggested that Western psychology is too culturally contaminated to be accepted prima facie by Muslims. To this extent, I agree with Badri (1979). I have argued, however, that a comprehensive psychology cannot be evolved simply from a culture-free experimental methodology and that Muslims should consider basing their understanding of the self on models that derived from *Qur’anic* teaching. I have proposed that it is both unnecessary and impractical to do this without reference to the models constructed by traditional Islamic scholars whose work as physicians and psychologists was imbued with Islamic understanding. I have presented a rudimentary model of the self (based on my understanding of such sources) which though doubtless in need of improvement points the way of understanding complex mental phenomena more comprehensively than is possible with most Western theories. It permits an absolute definition of mental health rather than one which is socially and culturally dependant. Its use by no means excludes the adoption of various theories and therapeutic techniques developed in the West, but enables the Muslim to be discriminating in how he uses this knowledge.

To return to the starting point of this paper, it is a model with which I personally feel comfortable within clinical practice and in helping me to give meaning to my own experience of self: it accords with my own sense of *Fitrah*.

I do not suggest that the model is complete: even less do I feel that the full range of diagnostic and therapeutic inferences from this model have been demonstrated. There are statements in *Qur’an* and *Hadith* referring to areas within the parameters of psychology (such as possession by *Djinn* and the use of dream analysis) which I have not even touched upon.

I hope, however, that this paper will provide a stimulus to constructive discussion and enables sketching both the framework for an Islamically appropriate psychology.

## Footnotes

1 I met Malik Badri after presenting this paper. Professor Badri said he shared my outlook, but when he published his book in 1979 he felt that most Muslim Psychologists were not ready to receive more than a limited critique of Western Psychology. Professor Badri refers to this in a preface to the new reprint of The Dilemma of Muslim Psychologists.

2 Birbeck College London.
